# Development and psychometric validation of caring leadership scale in nursing

**DOI:** 10.1371/journal.pone.0342972

**Published:** 2026-03-02

**Authors:** Fengjian Zhang, Lei Huang, Xiao Peng, Yang Fei, Juan Xu, Yilan Liu, Ning Zhang, Cheng Chen, Jie Chen

**Affiliations:** 1 Union Hospital, Tongji Medical College, Huazhong University of Science and Technology, Wuhan, China; 2 School of Nursing, Xinxiang Medical University, Xinxiang, China; 3 Department of Nursing, Yangtze University, Jingzhou, China; 4 College of Nursing, Florida State University, Tallahassee, United States of America; Sichuan Agricultural University, CHINA

## Abstract

**Background:**

Caring leadership plays a pivotal role in supporting nurses’ well-being, fostering patient recovery, and advancing organizational performance. Despite its recognized importance, a validated instrument for measuring caring leadership has been lacking in the literature.

**Objective:**

This study aimed to develop a Caring Leadership Scale and evaluate its validity, reliability, and practical applicability.

**Methods:**

Guided by a conceptual framework of caring leadership, scale items were initially generated through qualitative interviews, open-ended questionnaires, and a comprehensive literature review. The Delphi technique was used to refine these items. A pilot survey informed further modifications before a large-scale survey was conducted with 2,125 frontline nurses from six tertiary hospitals across China. Item screening was carried out using classical test theory, including assessments of item discrimination, item-total correlations, and internal consistency (Cronbach’s alpha). Content validity, factor structure, internal reliability, and acceptability were investigated using content validity indices, exploratory and confirmatory factor analyses, correlation coefficients, and descriptive statistics.

**Results:**

An initial pool of 40 items across five dimensions was developed. Following two Delphi rounds and preliminary testing, 39 items were retained. Item analysis led to the elimination of 12 items that did not meet predetermined criteria. The final scale consisted of 27 items across five dimensions, rated on a 5-point Likert scale. The content validity indices were strong, with item-level CVIs ranging from 0.90 to 1.00, a scale-level content validity index universal agreement (S-CVI/UA) of 0.89, and a scale-level content validity index average (S-CVI/Ave) of 0.99. Exploratory factor analysis (EFA) supported a well-defined five-factor structure, with common factor variances ranging from 0.849 to 0.949. Confirmatory factor analysis demonstrated good model fit (RMSEA = 0.078, CFI = 0.964, NFI = 0.958, TLI = 0.959, IFI = 0.964). The average variance extracted (AVE) ranged from 0.834 to 0.925, and composite reliability (CR) ranged from 0.965 to 0.989. The scale showed moderate correlations with job satisfaction (r = 0.463–0.484, *p* < 0.001). Internal consistency was high, with Cronbach’s alpha values between 0.962 and 0.993, and split-half reliability coefficients ranging from 0.955 to 0.982.

**Conclusion:**

This study presents the first validated instrument for measuring caring leadership in nursing. The final 27-item scale demonstrates robust psychometric properties, including excellent validity, reliability, and user acceptability. It offers a scientifically sound and practical tool for assessing caring leadership among nurse leaders.

## 1. Introduction

Caring is considered the essence of nursing [1]. This foundational principle extends beyond patient-provider interactions to the core of organizational management within healthcare [2,3]. Amid ongoing challenges, including increasing demands for high-quality care, constrained financial resources, and workforce shortages, a caring-based leadership approach is crucial for aligning stakeholder interests and promoting organizational excellence [3–5]. Therefore, caring leadership has become vital in cultivating a supportive work environment and promoting favorable patient outcomes.

Caring leadership is defined as a relationship of friendliness, reciprocal trust, mutual respect, and tenderness among the leader and followers, and is characterized by a distinct interpersonal orientation [3,6]. Caring leadership centers on building genuine relationships with employees, showing concern for others, and attentively recognizing individual differences, fostering greater team collaboration [7]. Caring leadership involves showing genuine warmth and concern for subordinates, being attentive to their needs, promoting involvement in group decision-making and open communication, and ensuring fair and equal treatment for all team members [8].

Caring leadership has been consistently associated with positive employee behaviors and improved organizational performance. Previous studies have demonstrated that leaders showing genuine concern for their staff increase job satisfaction [9,10] and encourage constructive work behaviors [11,12]. Moreover, caring leadership has been found to diminish employees’ perceptions of workplace bullying [13,14]. It also plays a vital role in shaping patient experiences, as nurses’ perceptions of caring leadership have contributed to higher patient satisfaction [15].

Although the significance of caring leadership is well acknowledged, its precise measurement remains challenging due to conceptual and methodological limitations in existing tools. For example, the commonly referenced Leadership Behavior Description Questionnaire (LBDQ) includes a ‘consideration’ subscale, often used as a stand-in for assessing caring leadership [16]. The subscale comprised 45 items across six dimensions: tolerance of uncertainty, allowance of autonomy, consideration, demand alignment, consolidation, and prediction. While this instrument offered an initial framework for understanding the behavioral traits associated with caring leadership and allowed for a more detailed exploration of caring behaviors, it falls short in a key aspect. The dimension of “consideration” reflects a broad leadership quality and does not capture the specific, relational nuances of “caring” as understood within nursing. This limitation restricts the questionnaire’s applicability in nursing management settings.

Within the nursing field, several tools have been developed based on established caring theories. The Caring Factor Survey-Caring of Manager (CSF-CM), a 10-item scale grounded in Watson’s Caring theory [17], has demonstrated good reliability in the USA [17], Slovenia [18], and mainland China [19], respectively. This tool offers a relatively concise format and allows for the quick assessment of caring behaviors in nursing leaders. However, its main limitation lies in the absence of a well-defined, multi-dimensional structure, which prevents a detailed analysis of the specific caring behaviors that managers either demonstrate strongly or lack. As a result, it falls short in capturing the full complexity of caring leadership. Similarly, the Caring Assessment Tool-Administration (CAT-admin), which is also grounded in Watson’s theory, was designed to evaluate caring behaviors using a 38-item scale [20].

In comparison to the CSF-CM, this scale incorporates three dimensions: respect, shared decision-making, and non-caring, offering a more detailed and targeted assessment of caring leadership among nursing managers. However, it is important to note that while “Human Respect” and “Shared Decision-Making” reflect core attributes of caring leadership, the “Non-Caring” dimension is designed to capture negative behaviors. This inconsistency, like the dimensions, leads to a lack of conceptual alignment, complicating both data analysis and the interpretation of findings. Similarly, Nyberg’s Caring Assessment Scale (NCAS), a 20-item instrument based on Ray’s theory of bureaucratic caring, also aims to evaluate the caring behaviors of nurse managers [21]. In its development, the tool directly transformed theoretical concepts into operational definitions and questionnaire items, maintaining a strong focus on the core constructs and preserving the instrument’s theoretical integrity. However, it currently lacks robust psychometric validation and has been infrequently cited or used in published research.

Although current instruments offer a foundation for evaluating caring leadership, they display conceptual and methodological shortcomings. Most are grounded in Western caring philosophies, which may limit their conceptual scope and cross-cultural relevance [19,22]. Furthermore, these tools primarily assess observable leader behaviors, overlooking distinct personal traits integral to caring leadership and its effectiveness. Furthermore, the limited integration of practical experience with theoretical frameworks hampers the effectiveness of these tools in real-world settings. As a result, organizations face challenges translating assessment outcomes into targeted, effective leadership development strategies, and the interpretation of results often lacks sufficient theoretical depth.

To address these limitations, particularly the need for a culturally relevant and structurally coherent assessment tool, this study did not rely on adapting existing Western theories. Instead, the scale was developed based on a conceptual model of caring leadership, constructed previously using Strauss and Corbin’s grounded theory methodology, drawing from empirical data collected from Chinese nurses. This model offers a context-specific framework that reflects the core attributes and multi-dimensional nature of caring leadership. Accordingly, the objective of this study was to develop and rigorously validate the Caring Leadership Scale, to provide a scientifically robust, reliable, and contextually appropriate tool for evaluating and promoting caring leadership in nursing practice.

## 2. Materials and methods

**Fig 1:**
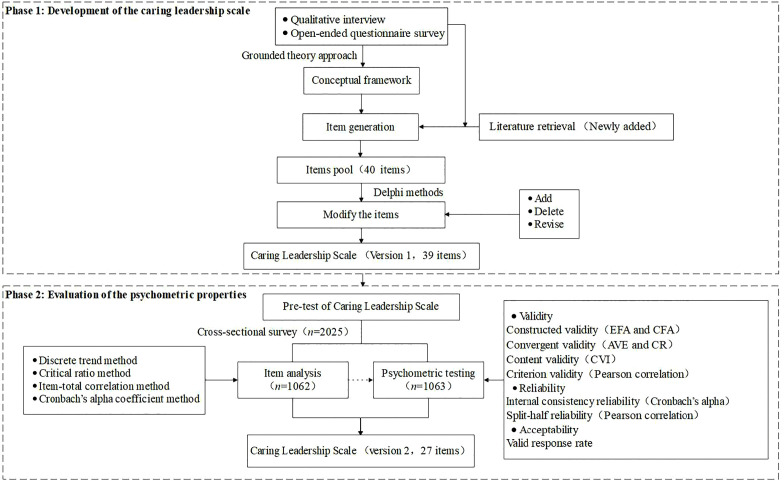
The procedure of caring leadership scale development and psychometric testing.

Fig 1 depicts the procedures of caring scale development and psychometric testing.

### 2.1. Phase 1: Development of the caring leadership scale

***Conceptual framework and item generation process*.** In a previous study, a caring leadership model was developed *via* Strauss and Corbin’s grounded theory approach that demonstrated the key elements of caring leadership [3]. The identified attributes served as the conceptual framework for the scale development process. Item generation followed two main approaches. First, key data were drawn from semi-structured interviews and open-ended questionnaires conducted in our previous research. Participants were asked two core interview questions: (1) What leader behaviors make you perceive someone as a caring leader? and (2) What traits or characteristics do you believe a caring leader should possess? Similarly, in the open-ended questionnaires, participants responded to: (1) What qualities in a nurse leader would make you consider her a caring leader? and (2) What attributes should a caring leader have?

Second, a comprehensive literature review was conducted to supplement and enrich the item pool. Searches were performed in PubMed, Web of Science, and the China National Knowledge Infrastructure (CNKI) using keywords such as (“caring leadership” OR “caring behavior” OR “considerate leadership”) and (“leader” OR “manager”), covering all literature up to June 30, 2021. Relevant studies were screened, and items related to caring leadership or the behaviors and traits of caring leaders were extracted. Inclusion criteria were: (1) studies describing caring behaviors or traits of leaders; and (2) studies that included assessment tools evaluating such behaviors or traits. Exclusion criteria included: (1) literature not published in English or Chinese, and (2) studies for which the full text could not be obtained after repeated attempts.

### Delphi methods

Experts were selected based on the following inclusion criteria: (1) a background in management, psychology, statistics, nursing, or a related discipline; (2) active involvement in research on humanistic caring or leadership, with at least one publication in a Chinese or SCI-indexed journal; (3) possession of an intermediate or higher professional title and a bachelor’s degree or above; (4) a minimum of five years of professional experience; and (5) provision of informed consent and voluntary participation in the study. Experts were excluded if: (1) they returned the consultation questionnaire after the designated deadline; or (2) their responses contained missing data that remained unresolved despite follow-up.

A purposive sample of 25 experts in nursing management, humanistic caring, psychology, and scale development was invited to participate in the Delphi process. All experts held at least a bachelor’s degree and intermediate or senior professional titles, with a mean working experience of 31.2 years. Each expert had published at least one article related to humanistic caring, leadership, or nursing management in peer-reviewed journals, ensuring both practical and academic expertise. Experts were purposively sampled from different institutions and disciplines to enhance the diversity and representativeness of the panel.

During the Delphi rounds, experts were asked to comment on the conceptual labels and allocation of items to dimensions. While no new factors were suggested, experts contributed to refining item wording and confirming the conceptual appropriateness of the five-factor framework. Moreover, experts and participants were also encouraged to comment on redundancy or ambiguity in item content. Several overlapping items were merged or removed at this stage. Eligible experts received the consultation questionnaire and were asked to assess each item in terms of relevance (1 = completely irrelevant, 4 = highly relevant) and importance (1 = very unimportant, 5 = very important) [23]. The items with the percentage of experts’ relevance score 3 or 4 < 80%, those with an average importance score ≤3 Points and a coefficient of variation (CV) ≥0.35 were considered for deletion [24]. Expert engagement was quantified by the response rate, defined as the proportion of experts who returned a valid questionnaire within the specified time frame divided by the total number of experts invited, multiplied by 100%. A response rate exceeding 80% was considered indicative of strong engagement. The basis of judgment (Ca) reflected the extent to which experts relied on theoretical analysis, practical experience, literature references, and subjective judgment in their evaluations. For each component, experts indicated whether their judgments were based on a strong, moderate, or weak foundation, and the corresponding weights were assigned as follows: theoretical analysis (0.4, 0.3, 0.2), practical experience (0.3, 0.2, 0.1), literature references (0.2, 0.1, 0.1), and subjective judgment (0.1, 0.1, 0.1), respectively. The Ca score for each expert was obtained by applying these weights to the expert’s selected levels across the four components and then combining the weighted component scores into a single overall judgment-basis score. Familiarity with the topic (Cs) reflected experts’ self-assessed familiarity with the scale content and was rated on a 5-point scale: very familiar (1.0), quite familiar (0.8), familiar (0.6), unfamiliar (0.4), and very unfamiliar (0.2). The Cs score for each expert was calculated by converting the selected category into its corresponding numeric value. Finally, the overall authority coefficient (Cr) was computed as the mean of Ca and Cs, and a Cr value above 0.70 was considered indicative of high expert authority [25].

### 2.2. Phase 2: Evaluation of the psychometric properties

***Sample and procedure.*** *Pre-test*: The initial version of the Caring Leadership Scale (Version 1) was administered to frontline nurses at a teaching hospital. To ensure participants had consistent and meaningful exposure to their immediate supervisor’s leadership style [3,26], the following inclusion criteria were applied: (1) active engagement in frontline nursing duties, (2) a minimum of two years of continuous service in their current department, and (3) voluntary consent to participate. Nurses were excluded if they were (1) undergoing training, departmental rotation, or orientation, (2) on extended leave exceeding six months, or (3) diagnosed with emotional or psychological disorders. The third exclusion criterion was evaluated through a self-reported item in the demographic section, which asked whether the participant had ever received a formal diagnosis of a cognitive or psychiatric condition from a healthcare provider. Questionnaires were distributed and collected individually by the researcher, who also documented participants’ feedback on the clarity and relevance of the items, as well as the time required to complete the scale.

*Formal survey.* Based on the economic development level [27], the study comprised registered nurses from six general hospitals in the eastern, central, and western regions of Mainland China, which included two hospitals in each area. Nurses were recruited through convenience sampling between December 30, 2021, and January 5, 2022, using the online survey platform Wenjuanxing (https://www.wjx.cn/). The inclusion and exclusion criteria remained consistent with those used in the pre-test phase. Following data collection, item analysis and psychometric evaluations were done sequentially. Item analysis was conducted using classical test theory, employing multiple approaches, namely, the discrete trend method, critical ratio method, item-total correlation analysis, and Cronbach’s alpha coefficient to determine whether specific items should be retained or removed [28].

Finally, the psychometric properties of the scale, including validity, reliability, and acceptability, were assessed using content validity index (CVI), factor analysis, correlation analysis, and descriptive statistics. The sample size was determined based on the requirements of these analytical methods. During the psychometric evaluation phase, a larger sample is generally recommended to improve the generalizability and stability of factor analysis results, with a sample size of 1,000 considered optimal [29]. In this study, exploratory factor analysis (EFA) and confirmatory factor analysis (CFA) were performed for factory analysis. Moreover, a 10% dropout rate was considered. Thus, at least 2200 questionnaires were required.

***Data analysis*.** The collected data were initially cleaned and then analyzed using SPSS 26.0 and AMOS 24.0 (IBM Inc., Armonk, NY); the demographic characteristics of the participants were analyzed with descriptive statistics.

***Item analysis.*** (1) *Discrete trend method.* The estimated standard deviation (SD) of item scores was used to assess the degree of dispersion, where SD < 0.80 suggested poor dispersion. (2) *Critical ratio method.* The total scale scores were arranged in ascending order, with the lowest 27% classified as the low-score group and the highest 27% as the high-score group. An independent sample t-test was then conducted to evaluate the discriminative ability of each item. Items yielding a p-value greater than 0.05 or a t-value less than 3.00 were considered for potential deletion due to insufficient discrimination. (3) *Item-total correlation method*. Pearson correlation was used to investigate the correlation between item and total scores, with *P* > 0.05, or a correlation coefficient <0.40 used for deleting items [30]. (4) *Cronbach’s alpha coefficient method.* Internal consistency reliability was assessed using Cronbach’s α. A significant increase in the overall reliability coefficient following the removal of an item suggested that the item might be poorly aligned with the rest of the scale, indicating substantial heterogeneity and warranting consideration for deletion [31].

***Psychometric test*** *Validity analysis.* (1) *Constructed validity*: The constructed validity was performed with EFA and CFA. Kaiser-Meyer-Olkin (KMO) sampling adequacy measures and Bartlett’s sphere test were adopted to check the data’s suitability for EFA [32]. During the EFA, principal component analysis (PCA) was employed to extract common factors, using Promax oblique rotation with a kappa value of 4. Items were removed if they failed to meet the following criteria: factor loading less than 0.4; cross-loading greater than 0.4 on multiple factors with a loading difference less than 0.2; communality below 0.2; fewer than three items within a factor; or if the item could not be categorized or interpreted. After confirming data normality, CFA was performed using maximum likelihood estimation (MLE) and bootstrapping (*n* = 5000). Model fit was evaluated using multiple indices, including the root mean square error of approximation (RMSEA), normed fit index (NFI), comparative fit index (CFI), tucker–lewis index (TLI), and incremental fit index (IFI). In general, RMSEA˂0.08, CFI, NFI, TLI, and IFI values of ≥0.9 indicate acceptable model fitness [33], suggesting a further revision of the model. (2) *Convergent validity*: The convergent validity was calculated after CFA. (3) *Discriminant validity*: Discriminant validity was examined using the Fornell–Larcker criterion, by comparing the square root of the AVE for each factor with its correlations with the other factors based on the CFA results. (4) *Content validity*: The content validity was reflected by the CVI, including item-level (I-CVI) and scale-level (S-CVI). Experts rated the relevance of each item on a 4-point scale (1 = not relevant to 4 = highly relevant). The item-level content validity index (I-CVI) was calculated as the proportion of experts rating an item as 3 or 4. The scale-level CVI universal agreement (S-CVI/UA) was computed as the proportion of items with I-CVI = 1.00, and the scale-level CVI average (S-CVI/Ave) as the average of all I-CVIs. In line with Lynn’s recommendations, I-CVI ≥ 0.78 and S-CVI/Ave ≥ 0.90 were considered acceptable [34,35]. (5) *Criterion validity*: The relationship between caring leadership and job satisfaction was investigated. Previous research has demonstrated a positive association between nurse leaders’ caring behaviors and nurses’ job satisfaction, indicating that caring leadership can improve overall work satisfaction among nurses. In this study, job satisfaction was assessed using a single-item measure rated on a 10-point scale (1 = extremely dissatisfied, 10 = extremely satisfied), asking, “How do you feel about your work in this hospital?”

*Reliability analysis.* The consistency of the scale was assessed through internal consistency reliability and split-half reliability. (1) *Internal consistency reliability*: Cronbach’s α was calculated to assess the scale’s reliability and dependability of each dimension. (2) *Split-half reliability*: The scale and each dimension were divided into two parts in odd and even order to calculate the split-half reliability.

*Acceptability analysis.* In this study, a valid response rate was used to evaluate the acceptability of the scale.

### 2.3. Ethical approval

Ethical approval for this study was obtained from ethics committee of Tongji Medical College of Huazhong University of Science and Technology (Approval Number: S137). Before completing the questionnaire, participants were presented with an online informed consent form that included “Yes” and “No” response options. Only those who selected “Yes” could proceed with the survey. All responses were collected anonymously, and the data were securely stored on a password-protected computer accessible only to the research team to maintain confidentiality.

## 3. Results

### 3.1. Development of the Caring Leadership Scale

#### 3.1.1. Development of the item pool.

Guided by the conceptual framework, an initial pool of 40 items was developed, structured around five dimensions: benevolence toward others (8 items), appreciation of uniqueness (7 items), facilitation of self-actualization (10 items), maintenance of mutual benefit (7 items), and motivation through charisma (8 items). Each item was rated using a 5-point Likert scale, ranging from 1 (strongly disagree) to 5 (strongly agree). The full list of items and their corresponding sources are provided in S1 File in S1 Data.

#### 3.1.2. Results of the Delphi study.

A consensus among experts was achieved through two rounds of the Delphi method. In the first round, 25 experts were invited, and 23 returned valid responses within the designated timeframe, yielding a response rate of 92.0%. The experts had a mean age of 51.96 years and an average of 31.22 years of professional experience; all held at least a bachelor’s degree, and 100.0% had senior professional titles. Detailed information about the experts consulted is available in S2 File in S1 Data. The authority coefficient was calculated at 0.91. The relevance ratings (scores of 3 or 4) ranged from 87.0% to 100.0%, I-CVI ranged from 0.87 to 1.00, average item scores fell between 4.43 and 5.00, and the CV ranged from 0.00 to 0.19.

In the second round, 23 consultation questionnaires were distributed, and 20 valid responses were received, resulting in an effective response rate of 86.96%. In this round, 90.0% to 100.0% of experts rated the items as relevant (scores of 3 or 4), I-CVI ranged from 0.95 to 1.00, average item scores were between 4.45 and 5.00, and the CV ranged from 0.00 to 0.15. Based on expert feedback and predetermined criteria, the items were revised accordingly through both Delphi rounds. As a result, a preliminary version (Version 1) of the 39-item scale was finalized, and the complete English versions of this scale is provided in S3 File in S1 Data.

### 3.2. Evaluation of the psychometric properties

#### 3.2.1. Pre-test.

A pilot test was conducted with 120 nurses recruited from multiple departments across a tertiary teaching hospital. Stratified sampling method was employed to ensure variation in age, years of experience, and departments. The average time taken to complete the questionnaire was 173 seconds, with a range of 90–314 seconds. No major comprehension issues were reported across different age, education, and experience groups, suggesting satisfactory face validity. Overall, they found the items to be clear, straightforward, easy to understand, and easy to respond to.

#### 3.2.2. Demographic characteristics in a formal survey.

In this study, a total of 2,442 nurses completed the online questionnaire, which included demographic information and the Caring Leadership Scale. After screening the data, questionnaires showing patterned responses (*n* = 250) or completed in an excessively short time (*n* = 67) were excluded. No participants reported emotional or psychological disorders. Ultimately, 2,125 valid responses were retained and randomly assigned for EFA (*n* = 1062) and CFA (*n* = 1063), the complete dataset is available in S4 File in S1 Data.

Among the valid participants, 47 were male and 2,078 were female. The average age was 32.49 years, with 68.85% being married. Most respondents held a bachelor’s degree (74.03%) and had an average of 10.47 years of work experience. A majority were employed at tertiary hospitals (85.46%) and held junior professional titles (78.77%). Participants represented various clinical departments, with 44.71% working in medical and surgical units. Most respondents reported a monthly income between 0.5 and 1.0 million yuan (54.45%). Detailed demographic information is provided in Table 1.

**Table 1:** Demographic data of participants in formal survey(*n* = 2125).

Characters	*n*	*%*	*M*	*SD*
Gender				
Male	47	2.21		
Female	2078	97.79		
Age (years)			32.49	6.50
< 30	866	40.75		
31 ~ 40	1049	49.37		
41 ~ 50	173	8.14		
≥ 51	37	1.74		
Marital status				
Unmarried	608	28.61		
Married	1463	68.85		
Divorced or widowed	54	2.54		
Education				
Postgraduate and above	33	1.55		
Bachelor’s degree	1573	74.03		
Secondary or advanced diploma	519	24.42		
Work Years (years)			10.47	6.97
< 10	1198	56.38		
11 ~ 20	742	34.91		
21 ~ 30	141	6.64		
≥ 31	44	2.07		
Title				
Senior	35	1.65		
Intermediate	416	19.58		
Junior	1674	78.77		
Hospital Level				
Tertiary hospital	1816	85.46		
Secondary hospital	309	14.54		
Work Department				
Medicine	528	24.85		
Surgery	422	19.86		
Obstetrics and Gynecology	200	9.41		
Pediatrics	191	8.99		
Outpatient and Emergency	198	9.32		
ICU	80	3.76		
Operation	117	5.51		
Oncology	48	2.26		
Geriatrics	27	1.27		
Orthopedics	61	2.87		
Others	253	11.91		
Average monthly income(CNY)				
Less than 5000	652	30.68		
5000 ~ 10000	1157	54.45		
More than 10000	316	14.87		

#### 3.2.3. Item analysis.

During the item analysis, the SD of the items ranged from 0.81 to 1.08, all exceeding the threshold of 0.80. The CV ranged from 0.18 to 0.26, also above the acceptable cutoff of 0.15. Independent samples t-tests revealed t-values between 30.591 and 37.378 (all > 3.00, *p* < 0.001), indicating strong item discrimination. The item-total correlation coefficients ranged from 0.833 to 0.950 (all > 0.80, *p* < 0.001), demonstrating strong correlations between each item and the total scale score. The overall Cronbach’s α coefficient was 0.995, and this value remained unchanged when individual items were deleted, suggesting high internal consistency across the scale. As shown in S5 File in S1 Data, all 1,062 respondents’ item analysis results met the required criteria; therefore, no items were removed. Detailed information on the relevant results is provided in S5 File in S1 Data.

### 3.3. Validity

#### 3.3.1. Structure Validity.

**(1) Exploratory factor analysis**

Before conducting the EFA, the KMO measure of sampling adequacy was 0.985, and Bartlett’s test of sphericity yielded a value of 54,044.508 (*p* < 0.001), indicating that the data were well-suited for factor analysis. EFA was performed using PCA, with the number of factors fixed at five and an oblique rotation (Promax) applied, based on the conceptual framework.

The analysis revealed that items Ab1, Ab2, Ab7, Ab8, Ac5, Ac6, Ad5, and Ad7 had factor loadings below 0.40 and were therefore removed. Moreover, items Aa5, Aa8, Aa9, and Ad4 showed cross-loadings greater than 0.40 on two factors, with differences in loading less than 0.20, and were also excluded. Although item Ad6 did not load on its initially assigned factor, it was retained because conflict resolution is a core component of caring leadership in our grounded theory model and is closely aligned with the characteristics of the factor on which it loaded. The decision was made after group discussion, balancing empirical and theoretical considerations.

After removing items that did not meet the criteria, the factor structure became more distinct. In the exploratory factor analysis, the number of factors was fixed at five in accordance with the previously developed conceptual model of caring leadership [36,37]. The resulting five-factor solution showed a clear and interpretable simple structure, with communalities for the retained items ranging from 0.849 to 0.949 and all factor loadings on their intended factors being high. The final factor loading matrix and communalities after item deletion are presented in Table 2. The resulting 27-item Caring Leadership Scale (Version 2) is shown in S3 File in S1 Data.

**Table 2:** Factor loading matrix with common factor variance after deletion of items.

Item	Factors					Common factor variance
	Factor1	Factor2	Factor 3	Factor 4	Factor5	
Ae6	0.878					0.936
Ae7	0.872					0.917
Ae8	0.850					0.915
Ae9	0.811					0.932
Ae5	0.789					0.925
Ae3	0.760					0.923
Ae2	0.701					0.892
Ae4	0.678					0.934
Ae1	0.548					0.875
Ad6	0.510					0.892
Aa4		0.929				0.853
Aa1		0.769				0.880
Aa2		0.722				0.905
Aa3		0.651				0.884
Aa6		0.510				0.856
Aa7		0.490				0.886
Ab4			0.799			0.930
Ab5			0.776			0.916
Ab6			0.600			0.849
Ab3			0.551			0.888
Ad1				0.830		0.927
Ad2				0.752		0.935
Ad3				0.419		0.902
Ac3					0.587	0.942
Ac4					0.586	0.932
Ac2					0.576	0.949
Ac1					0.490	0.933

**(2) Confirmatory factor analysis**

**Fig 2:**
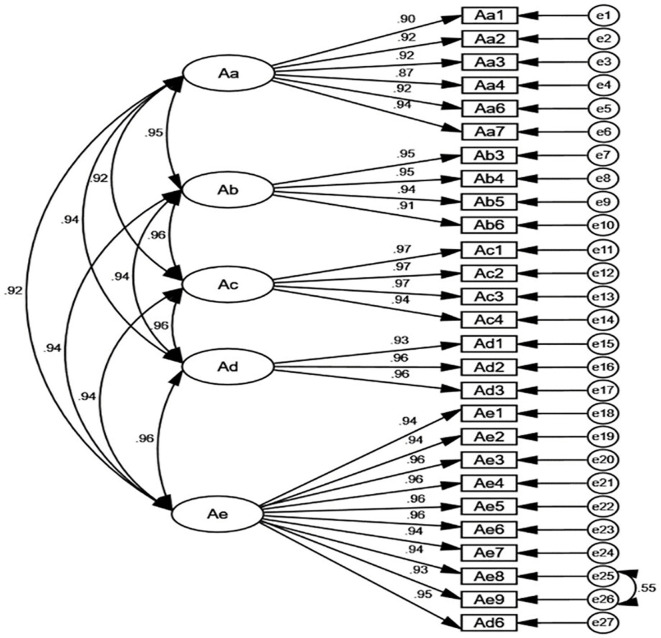
Standardized five-factor structural model of Caring Leadership Scale (n = 1063). Note: RMSEA = 0.078, CFI = 0.964, NFI=0.958, TLI = 0.959, IFI = 0.964.

A total of 1,063 questionnaires were used for CFA. Prior to CFA, univariate normality was evaluated for all items. Skewness values ranged from −0.1784 to 0.1425 (all |skewness| < 2), and kurtosis values ranged from 1.374 to 3.236 (all |kurtosis| < 7), indicating that the item distributions did not markedly deviate from normality. The five-factor model was estimated using the MLE method, with 5,000 bootstrap resamples and a 95% confidence interval. The initial model fit indices were: RMSEA = 0.084, CFI = 0.957, NFI = 0.952, TLI = 0.952, and IFI = 0.957, suggesting the need for model refinement. Modification indices suggested several pairs of error terms within the same factor that could be freely correlated. The largest index was for the residuals of two items within the same latent factor (errors e25 and e26; MI = 309.975). Because these items showed highly similar content, their residuals were allowed to covary. After appropriate modifications, the model fit improved, with updated indices as follows: RMSEA = 0.078, CFI = 0.964, NFI = 0.958, TLI = 0.959, and IFI = 0.964. The final standardized factor loading model obtained from the CFA is presented in Fig 2.

Overall, the final 27 items mapped well onto the five dimensions proposed in the grounded theory model of caring leadership, and the factor structure was consistent with the conceptual framework.

#### 3.3.2. Convergent Validity.

The average variance extracted (AVE) of each factor was suggested to be greater than 0.5, and the composite reliability (CR) should be greater than 0.7, which indicates a good convergent validity [32]. The AVE of the five dimensions was 0.834, 0.875, 0.925, 0.903, and 0.899, whereas the CR of the five dimensions was 0.968, 0.966, 0.980, 0.965, and 0.989, suggesting high convergent validity. The detailed results are available in S6 File in S1 File.

#### 3.3.3. Discriminant validity.

Discriminant validity was examined using the Fornell–Larcker criterion. The square roots of the AVEs (0.913–0.962) were compared with the interfactor correlations (r = 0.916–0.961). For factor Ac, the square root of the AVE exceeded its correlations with all other factors, thereby satisfying the criterion. In contrast, for several other factor pairs (e.g., Aa-Ab, Ab-Ac, Ac-Ad, Ad-Ae), the interfactor correlations were very close to or slightly higher than the corresponding square roots of the AVEs, indicating that discriminant validity among these dimensions was only partially supported and that certain facets of caring leadership are highly interrelated. The detailed results are available in S6 File in S1 File.

#### 3.3.4. Content validity.

Ten experts were re-invited to assess the items’ relevance. The results showed that the I-CVI ranged from 0.90 to 1.00, the scale-level content validity index universal agreement (S-CVI/UA) was 0.89, and the scale-level content validity index average (S-CVI/Ave) was 0.99. The detailed results are available in S6 File in S1 Data.

#### 3.3.5. Criteria validity.

Job satisfaction was used as the criterion variable to assess the criterion-related validity of the caring leadership scale. The correlation between overall caring leadership and nurses’ job satisfaction was 0.484 (*p* < 0.001). Furthermore, the correlations between the five dimensions of caring leadership and job satisfaction were 0.468, 0.482, 0.464, 0.474, and 0.463, respectively, all statistically significant at the *p* < 0.001 level. The detailed results are available in S6 File in S1 Data.

### 3.4. Reliability

Cronbach’s α of the scale level and its five dimensions ranged from 0.962 to 0.993, and the split-half reliability ranged from 0.955 to 0.982, all of which were >0.90, indicating the ideal internal consistency [32]. Table 3 depicts further information.

**Table 3:** Reliability coefficient of Caring Leadership Scale.

Dimension/Scale	Cronbach’s α coefficient	Split-half reliability
Benevolent to others	0.967	0.955
Appreciate the uniqueness	0.964	0.963
Facilitate self-actualization	0.980	0.977
Maintain mutual benefit	0.962	0.964
Motivate with charisma	0.989	0.982
Total scale	0.993	0.974

### 3.5. Acceptability

This study collected 2,442 responses from six general hospitals, reflecting a strong participation rate. After removing invalid questionnaires, 2,125 valid responses were retained, yielding a valid response rate of 87.0%, indicating that the scale had a satisfactory level of acceptability among participants.

## 4. Discussion

### The significance of the scale

Caring leadership is essential for effective management and organizational excellence, particularly within healthcare settings. However, previous studies have lacked appropriate tools to evaluate this leadership style. This study developed the first Caring Leadership Scale grounded in a culturally relevant conceptual model derived from previous research [3], and confirmed the validity of the five core attributes identified earlier. The scale addresses a significant gap by offering a reliable and evidence-based instrument to assess caring leadership among nurse leaders. Given the importance of caring leadership in both Chinese cultural contexts [38,39] and healthcare institutions [4]. The development of this scale enables broader investigation into its organizational impact and value.

### The scientificity of the scale

The development of the Caring Leadership Scale adhered to established scale development procedures to ensure scientific rigor [40]. Items were initially generated from interviews, open-ended questionnaires, and a review of relevant literature. These items were then organized into dimensions aligned with the conceptual framework of caring leadership, therefore ensuring the scale’s relevance and applicability within the Chinese cultural context [41]. Two rounds of Delphi consultation with multidisciplinary experts served as a formal quality-control step to improve item clarity and relevance. The experts’ strong academic and practical backgrounds, together with high participation across rounds, suggest that the refinement process was credible and that the final items likely represent a shared professional understanding of caring leadership. Across both rounds, the gradually increasing content validity demonstrating a clear tendency toward expert consensus [35]. Overall, this multi-source and iterative approach supports the scale’s content representativeness and practical applicability.

From a psychometric perspective, the available evidence suggests that the scale functions as a theoretically meaningful and structurally coherent measure. Dimensionality was evaluated using a two step strategy with independent samples, whereby exploratory findings were subsequently examined through confirmatory modeling. This approach reduces the likelihood that the observed structure is sample specific and supports interpretation of the scale as multidimensional. Evidence of convergent validity further indicates that items within each dimension reflect a common underlying construct. However, discriminant validity was not fully supported under strict statistical criteria, as correlations among several dimensions were very high and were comparable to or exceeded conventional benchmarks for adequate separation [42]. This pattern is theoretically plausible because the five dimensions represent closely related facets of a higher order construct, caring leadership, and such behaviors may co occur in routine nursing management practice. In addition, the cross sectional single source self report design may have inflated associations due to common method variance and shared response tendencies [43]. Collectively, these findings suggest that the scale captures a coherent overarching construct, while also indicating that further work is needed to strengthen the distinctiveness of some dimensions.

Beyond internal structure, the scale demonstrated theoretically expected associations with an external criterion, supporting its potential value for research and practice. Positive relationships between caring leadership scores and nurses job satisfaction are consistent with prior evidence, suggesting that the scale reflects leadership behaviors relevant to nurses work experiences [9,10]. Reliability evidence indicates strong score consistency, and the magnitude was comparable to or higher than that reported for established caring-related instruments such as the CSF-CM (α = 0.89) and CAT-adm (α = 0.942), supporting its use in assessment and feedback. Importantly, the strong reliability may also reflect the scale’s cultural grounding. Unlike many Western tools that primarily emphasize relational support, communication, and general consideration, the present scale incorporates culturally salient elements such as benevolence toward others and motivation through charisma, which align with moral-oriented leadership expectations and role-model influence commonly emphasized in Chinese organizational contexts. These culturally embedded dimensions may help explain why the scale captures a coherent construct in this population and may provide incremental explanatory value when assessing caring leadership in Chinese nursing management settings.

Nevertheless, very high internal consistency may also raise the possibility of item redundancy [44]. Such findings can reflect a well defined construct and a coherent item set, particularly when items are informed by qualitative data and refined through iterative expert consultation, but should be interpreted alongside additional diagnostics. Future studies should evaluate redundancy more directly using inter item correlations and omega coefficients, examine alternative measurement structures such as second order confirmatory factor analysis or bifactor models, and assess discriminant validity using complementary indices such as the heterotrait monotrait ratio.

### The practicability of the scale

The finalized Caring Leadership Scale comprises five dimensions and 27 items. With a moderate item count and clear, easy-to-follow instructions, the scale demonstrates strong usability and practical relevance. Compared to existing assessment tools, its items capture behaviors and characteristics closely aligned with caring leadership, making it well-suited to inform training and development in future studies. In healthcare settings, the scale holds considerable practical value. At the organizational level, it can be used as a diagnostic tool to assess leadership strengths and pinpoint areas that require targeted intervention across departments. It also serves as a reliable pre- and post-assessment instrument for evaluating the impact of leadership development initiatives, such as workshops, mentoring, or training programs. At the individual level, the scale’s multi-dimensional design enables detailed, actionable feedback rather than relying on a single composite score. Nurse leaders can use their dimension-specific profiles to identify personal strengths and improvement areas, thus facilitating tailored coaching and informing the development of individualized professional growth plans for sustained leadership advancement.

## 5. Limitations

This study has several limitations. First, the scale was validated using ratings from frontline nurses in several tertiary hospitals within one country, mainly female, and with convenience online sampling. Although this reflects the actual structure of the nursing workforce, it may limit the generalizability of the findings to male nurses, other types of institutions, regions, and healthcare systems. Future studies should use more rigorous sampling strategies, include more male nurses and nurse leaders, and examine measurement invariance across key subgroups. Second, the cross-sectional, anonymous online design precluded assessment of test-retest reliability and may have introduced selection and response bias. Because the data were self-reported and collected in an uncontrolled environment, common method bias cannot be ruled out. In addition, discriminant validity was not fully supported, and the high correlations among some dimensions suggest potential overlap. Longitudinal and multi-source designs are recommended to evaluate temporal stability and strengthen construct distinctiveness. Third, criterion-related validity was examined only with a single-item measure of job satisfaction, and the scale was developed within a specific cultural context. Future research should include additional outcomes and conduct cross-cultural adaptation and validation to further test the robustness and applicability of the Caring Leadership Scale.

## 6. Conclusion

This study developed and validated a culturally grounded Caring Leadership Scale for nursing. The 27-item, five-dimension instrument demonstrates strong evidence of validity, reliability, and acceptability, supporting its use for leadership assessment and development in nursing management. By operationalizing culturally salient facets such as benevolence and charisma, the scale extends existing caring-related measures and offers a more context-sensitive evaluation framework. Future studies should confirm its measurement invariance and predictive utility across diverse settings and populations.

## Supporting information

S1 Data (RAR)
